# Dietary Fibre and Organic Acids in Kiwifruit Suppress Glycaemic Response Equally by Delaying Absorption—A Randomised Crossover Human Trial with Parallel Analysis of ^13^C-Acetate Uptake

**DOI:** 10.3390/nu14153189

**Published:** 2022-08-03

**Authors:** John Monro, Suman Mishra, Halina Stoklosinski, Kerry Bentley-Hewitt, Duncan Hedderley, Hannah Dinnan, Sheridan Martell

**Affiliations:** 1 The New Zealand Institute for Plant and Food Research Limited, Private Bag 11600, Palmerston North 4442, New Zealand; suman.mishra@plantandfood.co.nz (S.M.); halina.stoklosinski@plantandfood.co.nz (H.S.); kerry.bentley-hewitt@plantandfood.co.nz (K.B.-H.); duncan.hedderley@plantandfood.co.nz (D.H.); hannah.dinnan@plantandfood.co.nz (H.D.); sheridan.martell@plantandfood.co.nz (S.M.); 2 Riddet Institute, University Avenue, Fitzherbert, Palmerston North 4474, New Zealand

**Keywords:** kiwifruit, glycaemic response, absorption, dietary fibre, organic acids, ^13^C acetate

## Abstract

Non-sugar components of kiwifruit reduce the amplitude of the glycaemic response to co-consumed cereal starch. We determined the relative contribution of different non-sugar kiwifruit components to this anti-glycaemic effect. Healthy participants (*n* = 9) ingested equal carbohydrate meals containing 20 g starch as wheat biscuit (WB, 30 g), and the sugar equivalent of two kiwifruit (KFsug, 20.4 g), either intrinsic or added as glucose, fructose and sucrose (2:2:1). The meals were WB+KFsug (control, no non-sugar kiwifruit components), WB + whole kiwifruit pulp (WB+KF), WB + neutralised kiwifruit pulp (WB+KFneut), WB + low-fibre kiwifruit juice (WB+KFjuice) and WB+KFsug + kiwifruit organic acids (WB+KFsug+OA). All meals were spiked with 100 mg sodium [1-^13^C] acetate to measure intestinal absorption. Each participant ingested all meals in random order. Blood glucose and breath ^13^CO_2_ were measured at ingestion and at 15 min intervals up to 180 min. Compared with WB+KFsug, whole kiwifruit pulp (WB+KF) almost halved glycaemic response amplitude (*p* < 0.001), reduced incremental area under the blood glucose response curve (iAUC) at 30 min (peak) by 50% (*p* < 0.001), and averted late postprandial hypoglycaemia. All other treatments suppressed response amplitude half as much as whole kiwifruit and averted acute hypoglycaemia, with little effect on iAUC. Effects on ^13^CO_2_ exhalation paralleled effects on blood glucose (R^2^ = 0.97). Dietary fibre and organic acids contributed equally to the anti-glycaemic effect of kiwifruit by reducing intestinal absorption rate. Kiwifruit flesh effectively attenuates glycaemic response in carbohydrate exchange, as it contains fructose, dietary fibre and organic acids.

## 1. Introduction

Kiwifruit are widely regarded as very nutritious, with a high content of Vitamin C [1]. In common with most ripe fruit, they also contain a high concentration of fruit sugars [2], which are often regarded as a challenge to blood glucose management. However, in a diet in which carbohydrate intakes are prudently managed, kiwifruit have been shown to contribute to glycaemic control [3], for two main reasons. Firstly, the available carbohydrate in kiwifruit is about 50% fructose, which has a low glycaemic index (GI) of about 22 [4]. Equicarbohydrate partial exchange of kiwifruit for foods containing highly digestible starch, which can have a GI approaching that of glucose [4], may therefore substantially reduce glycaemic impact [3,5]. Secondly, components of kiwifruit, apart from sugars, add to the anti-glycaemic effect [6]. The combination of kiwifruit sugars and properties of digested kiwifruit remnants in the gut may cause a substantial lowering of glycaemic response to a co-consumed starchy cereal [3], an effect that has been noted in other fruit [7].

Individual components of kiwifruit could reduce glycaemic impact in a number of ways. The slurry of cell wall remnants produced when kiwifruit are digested surrounds other foods and retards processes involved in the glycaemic response, such as glucose diffusion, luminal mixing and digestion [8]. Organic acids, which are a quantitatively important component of kiwifruit, may inhibit salivary amylase digestion of starchy foods in the stomach by lowering the pH [9,10]. They may also delay the release of gastric chyme to the duodenum [11], as the exceptionally strong buffering capacity of kiwifruit organic acids [6] requires prolonged gastric production of HCl to reduce pH to target, and/or because the arrival of low pH, highly buffered chyme in the duodenum leads to activation of the enterogastric reflex, delaying gastric emptying [12].

Which components of kiwifruit are responsible for the lowering of glycaemic impact beyond that achieved by simple substitution of fruit sugar for starch has not yet been examined in vivo. It is, however, important that there is some understanding of the mode of action of a fruit that is widely eaten for its health benefits and is becoming an economically important crop in number of countries. Such knowledge will be important in developing new cultivars and new products for their health benefits, in giving substance to marketing messages, and in understanding the nutritional effects of consuming kiwifruit. And beyond kiwifruit, it will improve our understanding of the role of fruit generally in managing glycaemic response.

The ability of a fruit preload to suppress glycaemic response has been reported [13], and the anti-glycaemic effects of organic acids [10] and dietary fibre [14], have been known for some time. However, the relative contributions of the two fruit components within a single fruit to the overall effect, through effects on absorption, has not been determined, as far as the authors are aware.

In this report, we present results of a study in which participants consumed a cereal source of glycaemic starch accompanied by either whole kiwifruit, kiwifruit sugars, kiwifruit organic acids, neutralised kiwifruit or reduced-fibre kiwifruit juice, in amounts equivalent to their content in two kiwifruit. The aim was to quantify the role of different kiwifruit macro-components in the net ability of kiwifruit to suppress glycaemic response to co-consumed cereal starch, by gut-level effects. As confirmation of gastrointestinal effects of kiwifruit on absorption, an extrinsic stable isotope marker (sodium acetate [1-^13^C]) used in studies of gastric emptying [15] was included in each meal, and its absorption tracked as breath ^13^CO_2_, in parallel with blood glucose, insulin and appetite responses.

## 2. Materials and Methods

### 2.1. Meal Ingredients

Kiwifruit pulp was prepared from ripe, ready-to-eat kiwifruit (Actinidia chinensis var. deliciosa ‘Hayward’) supplied by Zespri International, Tauranga. The kiwifruit were peeled, the hard apex of the core removed, and the flesh chopped to a coarse pulp for 10 s in a Halde food processor.

Wheaten breakfast cereal (Weet-Bix^®^) was bought from a local supermarket. WeetBix™ was used as the cereal starch-based food because it has a high content of readily digested starch (64.2%) and a low sugar content (2.8%), with the macro-nutrient balance made up of fat (1.4%), protein (12%) and dietary fibre (10.1%). Glucose, fructose and sucrose were obtained from Davis Food Ingredients, Palmerston North, New Zealand, and malic acid, citric acid and ascorbic acid from ‘Simply Brewing’ brewing suppliers, Palmerston North.

Sodium [1-^13^C] acetate (99% enriched) was obtained from Cambridge Isotope Labs, USA. The acetate is hydrophilic, poorly absorbed in the stomach and rapidly metabolised after intestinal absorption to ^13^CO_2_, which is detected in breath and used as a valid and reliable non-invasive method for measuring gastric emptying [15].

### 2.2. Food Analyses

*Carbohydrate*: Kiwifruit pulp (80 mL) was adjusted to pH 6.5 with NaOH before adding 5 mL 1% pancreatin, 0.1 mL amyloglucosidase, and 0.1 mL invertase. The pulp was made to 200 mL with 0.1 M maleate buffer pH 6.5 and digested for 60 min at 37 °C, before 1 mL samples were taken into 4.0 mL absolute ethanol, mixed, allowed to stand 30 min, and centrifuged (3000 g). Sugars in the ethanolic supernatant were measured using the dinitrosalicylic acid reducing sugar procedure [16].The analyses showed that the kiwifruit pulp contained a total available carbohydrate content of 10.2 ± 0.12 g (mean ± SD) per 100 g of kiwifruit flesh, on which the intake of 20.4 g per 200 g of pulp (Table 1) was based.

*Organic acids*: The quantity of the organic acids required was determined from a combination of the titratable acidity and proportions of organic acids typical of kiwifruit (New Zealand Food Composition Database. Available online: http//www.foodcomposition.co.nz). The organic acids were titrated with 1 M. NaOH. In five determinations, 20.7 ± 0.44 mL of 1.0 M NaOH was required to neutralise 100 g of kiwifruit pulp. Based on the proportion of the organic acids in kiwifruit, the organic acid content of the sample was calculated to be: citric acid 970 mg, malic acid 260 mg, quinic acid 780 mg and ascorbic acid 85 mg. As quinic acid for human consumption was unavailable, it was replaced by a mixture of citric and malic in the same proportions as in the fruit (970:260). Taking into account that citric acid is tribasic, malic acid is dibasic, and quinic acid monobasic, the quinic acid contributed only about 10% to total acid equivalents. The final mixture used, as reasonably equivalent to the organic acids in 200 g kiwifruit pulp, was a mixture of citric acid 2.35 g, malic acid 0.63 g, and ascorbic acid 0.17 g.

**Figure A1:**
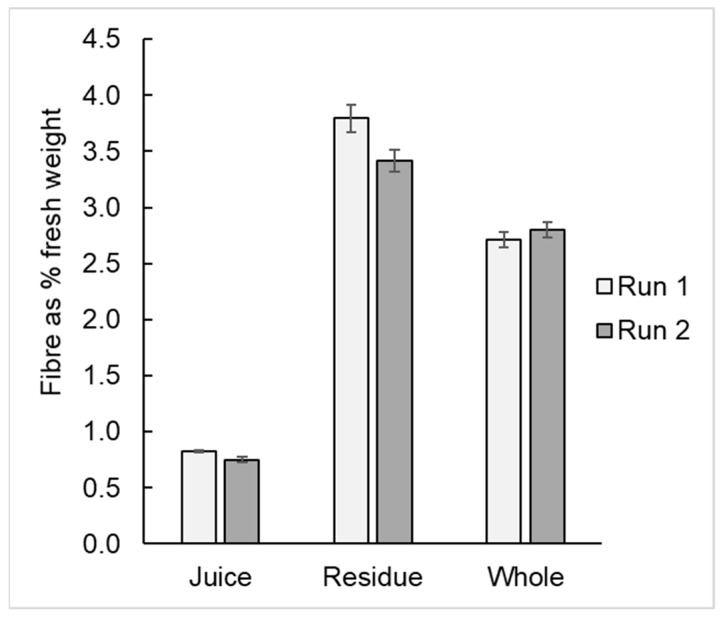
Dietary fibre content of expressed kiwifruit juice, of the residue from pressing and of the unpressed kiwifruit pulp.

*Dietary fibre*: Dietary fibre was determined in kiwifruit pulp, juice and pressed kiwifruit residue as the 80% ethanol-insoluble residue remaining after a static in vitro gastrointestinal digestion (Appendix A, Figure A1). Samples (10 g) were weighed in duplicate into 50 mL Falcon tubes. The contents were adjusted to pH 3.0, 1 mL of 10% pepsin solution was added and the contents were mixed. After 30 min incubation at 37 °C, the tubes were adjusted to pH 6.5 with 1 M NaOH, made to 20 mL, and 1.0 mL of 1% pancreatin and 0.1 mL amyloglucosidase added. The tube contents were mixed, and the tubes incubated for a further 30 min at 37 °C before freezing and freeze-drying. The freeze-dried material was crushed to a powder in situ with a glass rod, rehydrated in 10 mL water, and 40 mL ethanol added to achieve an 80% ethanol extraction. The dispersion was centrifuged, the supernatant discarded and the pellet resuspended in 80% ethanol before again centrifuging. The final pellet was rinsed with acetone and dried at 80 °C under vacuum before weighing as dietary fibre.

### 2.3. Preparation and Serving of Meals

Each participant consumed all of the meals detailed in Table 1. All meals contained:

- 30 g WeetBix™ (two biscuits) to provide a dose of 20.1 g of available carbohydrate, of which 96% was cooked starch;
- 20.4 g of kiwifruit sugars, either intrinsic to the fruit (Meals 2, 3, 4) or added as a mixture of glucose, fructose and sucrose (2:2:1) (Meals 1 and 5);
- 100 mg sodium acetate [1-^13^C, 99%], added at time of ingestion of the meal;
- Enough water to adjust all meals to a volume of 252 mL.

*Meal 1.* WeetBix™ + kiwifruit sugars (WB+KFsug).

Meal 1 was a control, containing all of the digestible carbohydrate but no non-sugar kiwifruit components. Based on the above analysis of kiwifruit sugars (10.2 ± 0.12 g/100 g pulp), 20.4 g lots of a mixture of glucose, fructose and sucrose (2:2:1) were weighed into individual specimen jars and stored at room temperature until consumed. For ingestion, each allocation was dissolved in 200 mL water and added to the WeetBix™.

*Meal 2.* WeetBix™ plus kiwifruit pulp (WB+KF).

The pulp (200 mL) was poured into sterilised individual freezer-proof containers with lids, snap frozen and stored at −20 °C until required.

*Meal 3.* WeetBix™ + Neutralised kiwifruit pulp (WB+KFneut).

The pulp was neutralised to pH 7 using food grade NaOH (1.0 M) (Ecochem Limited). The neutralised pulp (200 mL + 20.7 mL 2 M NaOH) was poured into sterilised individual freezer-proof containers with lids, snap frozen and stored at −20 °C until required.

*Meal 4.* WeetBix™ + kiwifruit juice (WB+KFjuice).

Kiwifruit juice was prepared by pressing the pulp in a basket juice press lined with 100 µm mesh nylon monomesh fabric (Filtercorp International Limited, Auckland, New Zealand). A subsample was taken for dietary fibre analysis (Appendix A Figure A1). For each participant, 194 mL juice (equivalent to 200 mL pulp with fibre present) was poured into sterilised individual freezer-proof containers with lids, snap frozen and stored at −20 °C until required.

*Meal 5*. WeetBix™ plus kiwifruit sugars plus kiwifruit organic acids. (WB+KFsug+OA).

**Figure A2:**
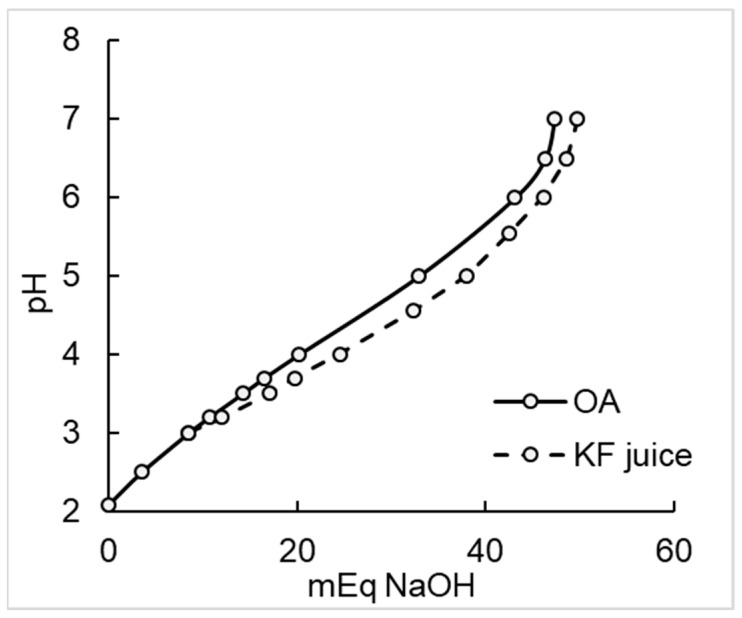
Titration of a single allocation of kiwifruit juice (294 mL, initial pH 3.3) and the organic acids (OA) mixture showed that OA adjusted to pH 3.3 closely replicated the kiwifruit juice in its neutralisation profile.

Citric acid (35.3 g), malic acid (9.46 g) and ascorbic acid (2.55 g) were dissolved in 200 mL water and adjusted to pH 3.3 with food grade NaOH (1.0 M, about 200 mL). The solution was made to 750 mL with water, and a dose of 50 mL allocated to each participant, to be consumed with 30 g WeetBix™ and 20.4 g of kiwifruit sugar mixture (Table 1). Titration curves of 200 g kiwifruit pulp and a single organic acid dose agreed well (Appendix A, Figure A2).

*Serving:* The frozen samples were thawed in a microwave without heating and added to the WeetBix™ before serving. For test Meals 1 and 5, the sugars and organic acids were served with WeetBix™ and the specified amount of water (Table 1). Acetate [1-^13^C] (100 mg) was mixed with each test meal just before serving.

### 2.4. Human Intervention Study

The human intervention study was approved by the Human and Disabilities Ethics Committee of the New Zealand Ministry of Health, and the trial was registered with the Australia New Zealand Clinical Trials Registry (Trial ID: ACTRN12620001225909). Meals prepared in house (that is, the frozen kiwifruit samples) were tested for microbial activity by the Plant & Food Research Analytical Lab (Auckland, New Zealand). Safety and preparation of food was approved by the Plant & Food Research Food Safety Committee.

The trial was run as a non-blinded, randomised, repeated measures study as it was not possible to blind the subjects to the differences between meals. Randomisation was achieved using a Williams-Latin square.

*Recruitment and screening*: Eleven healthy volunteers (18–40 y) were recruited using a flyer and email that briefly described the study, with pre-screening by phone. Selection of participants followed standard procedures involving anthropometric measurements and assessment of general health and glucose tolerance. Exclusion criteria were: glucose intolerance—any history of diabetes or evidence of glucose intolerance in preliminary tests of fasting blood glucose and HbA1c; allergic to or intolerant of kiwifruit and wheat; taking any antacids, laxatives or supplements; and recent ill health. Eleven subjects were recruited because 10 is the minimum number specified by the ISO method (ISO2664212010) for determination of glycaemic index and is typical of studies comparing glycaemic responses to foods. All subjects completed an informed consent form. Two subjects withdrew because of inconvenience of participating.

*Sampling procedure*: In preparation for each testing session, participants were asked to fast from 10:00 p.m. the night before a test, with water consumption not restricted, consume a similar moderate meal of their preference the evening before each test session to promote within-subject consistency, avoid strenuous physical activity and refrain from smoking or consuming alcohol the evening before a test and the morning of a test. At least 48 h was allowed between consecutive testing sessions. There was no restriction on foods naturally enriched in ^13^C.

**Figure 1:**
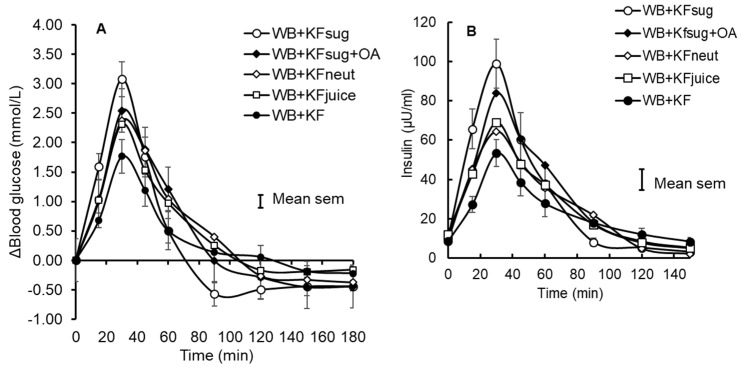
Incremental blood glucose (**A**) and insulin (**B**) responses to WeetBix™ (WB) in the presence of kiwifruit components: kiwifruit sugars (KFsug), kiwifruit sugars plus organic acids (KFsug+OA), neutralised kiwifruit pH 7 (KFneut), kiwifruit juice (KFjuice) and whole kiwifruit (KF). For clarity, error bars are attached to the WB+KFsug and WB+KF curves only (Means ± SEM).

On each test day, the volunteers were seated and asked to remain so for the duration of the test. On arrival, the participants were asked to relax for 15 min before two baseline fasting blood sugar measurements were made at −5 and 0 min. Participants were then given a test meal and instructed to consume all of it within 10 min. Blood glucose testing was timed from the start of food consumption. Finger prick sampling of capillary blood was at 15 min intervals in the first hour and then at 30 min intervals until 180 min had elapsed. Blood samples were thus collected at −5, 0 (baseline), 15, 30, 45, 60, 90, 120, 150, and 180 min (Figure 1). Blood glucose was measured immediately using a HemoCue^®^ blood glucose meter.

### 2.5. Outcome Analyses

*Blood glucose*: Blood glucose was measured immediately after sampling by finger-prick capillary blood analysis, using a HemoCue^®^ blood glucose meter.

*Insulin*: Finger prick blood was drawn into a Lithium Heparin (LH) microvette tube (CB 300 LH, Sarstedt AG & Co., Numbrecht, Germany), capped, inverted and stored on ice for a maximum of 1 h before centrifuging. Tubes were centrifuged at 2000× *g* for 15 min at 4 °C. Two 20 μL samples of the plasma were used to determine insulin by the assay procedure of the Human Insulin ELISA kit supplied by EMD Millipore Corporation (Cat. no. EZHI-14K). Absorbance was read at 450 nm on a FLUOStar Optima^®^ Plate reader (BMG Labtech, Victoria, Australia). A four-parameter logistic (4PL) standard curve was fitted for each ELISA plate and used to calculate human insulin concentrations (µU/mL) of the unknown plasma samples.

*Sodium acetate [1-^13^C] absorption*: Breath samples were collected in 12 mL Exetainer^®^ tubes (coated glass vials for ^13^C, Labco, UK) at baseline (0 min) and every 15 min for 3 h. The cap was removed from the vial, and a breath straw (product no. VP116/C, Labco, UK) was placed in it, ensuring that the tip of the straw extended to the bottom of the vial. The participant took two to three breaths to clear the lungs, inhaling and exhaling fully, and with the last exhalation gently blown through the straw into the vial while slowly withdrawing it from the vial. The cap was immediately replaced, to seal the vial.

The ^13^CO_2_ breath analysis was performed by the University of Waikato Stable Isotope Unit, using a Dumas elemental analyser (Europa Scientific ANCA-SL) interfaced to an isotope mass spectrometer (Europa Scientific 20-20 Stable Isotope Analyser) (Sercon Ltd., Cheshire, UK).

*Analysis of Breath ^13^CO_2_*: Atom percent excess ^13^C values were converted to Delta ^13^C values for analysis. The initial (T = 0) background value of each individual was subtracted from their subsequent values, and the incremental Delta ^13^C values were used in the analysis of treatment effects.

**Figure 2:**
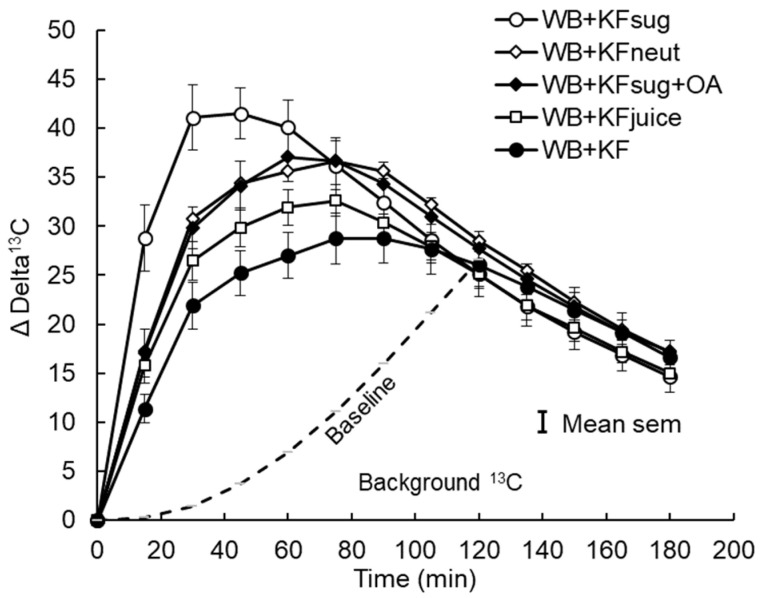
Incremental ^13^C release in breath CO_2_ after consuming sodium [1-^13^C] acetate in meals containing WeetBix™ (WB) and various kiwifruit components: kiwifruit sugars (KFsug), kiwifruit sugars plus organic acids (KFsug+OA), neutralised kiwifruit pH 7 (KFneut), kiwifruit juice (KFjuice) and whole kiwifruit (KF). The baseline (background) ^13^C release from ^13^C accumulated in body tissues (Poly. (BL)) is adjusted to the point where differences between treatments are no longer evident (120 min), and it is assumed that absorption was almost complete. Equation of baseline: y = 0.0017x^2^ + 0.0186x − 0.3601 (see Appendix A, Figure A3).

**Figure 3:**
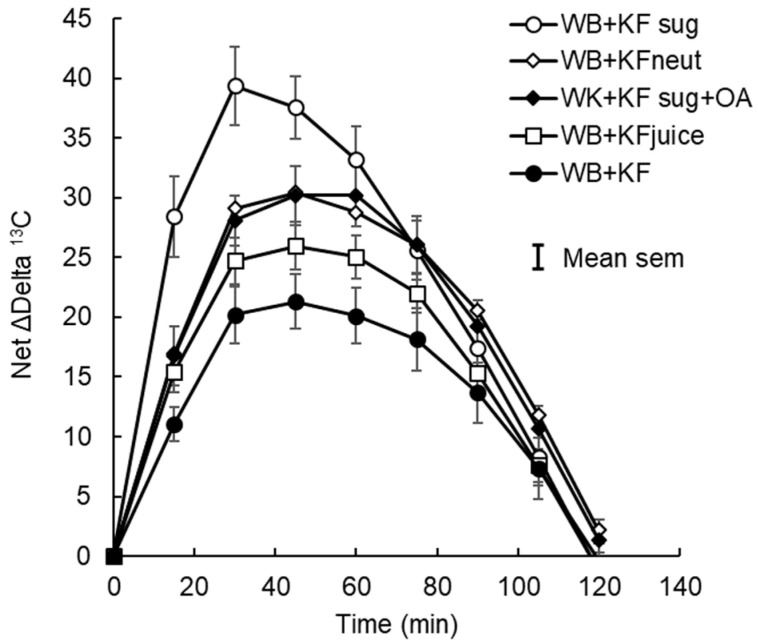
Net incremental breath ^13^C after subtraction of cumulative baseline ^13^C. Sodium [1-^13^C] acetate was ingested in meals containing WeetBix (WB) with kiwifruit sugars (KFsug), neutralised kiwifruit (KFneut), KFsug plus organic acids (OA), kiwifruit juice (KFjuice), or entire kiwifruit pulp (KF). The curve replicates glycaemic responses (Figure 1) but without the compressive effect of insulin.

**Figure 4:**
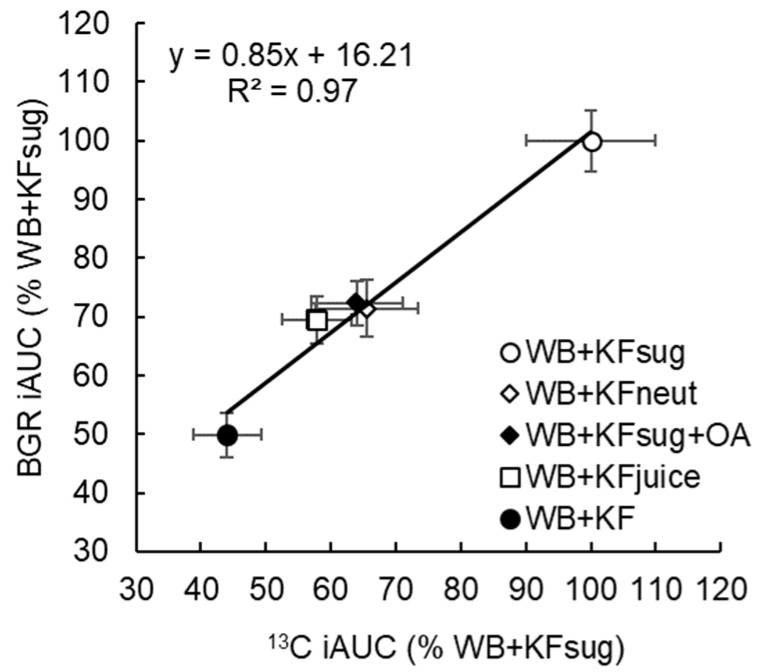
Cumulative incremental area under the curve of breath ^13^CO_2_ response (^13^C iAUC) and the blood glucose response (BGR iAUC) at 30 min (time of the glycaemic response peak) after ingestion, expressed as a percentage of WB+KFsug (100%). The meals contained WeetBix™ (WB), sodium [1-^13^C] acetate and various kiwifruit components: kiwifruit sugars (KFsug), neutralised kiwifruit pH 7 (KFneut), kiwifruit sugars plus organic acids (KFsug+OA), kiwifruit juice (low fibre) (KFjuice) and whole kiwifruit (KF). (Means ± SEM).

Because all subjects consumed all treatments, no adjustment was made for body conformation in calculating breath ^13^C. In the analysis of breath ^13^CO_2_, allowance was made for the effect of turnover of ^13^C accumulated in the body on the ^13^C background and its obscuration of absorption differences. This was done by adjusting the Delta ^13^C baseline from zero at the time of ingestion, to the point where the mean postprandial differences between treatments in breath ^13^C had disappeared but ^13^C output was still high compared with T = 0 (Figure 2). Because of the large differences between treatments at 30–40 min (Appendix A Figure A4), we assumed that disappearance of the differences at 120 min indicated that absorption was almost complete, so the delta ^13^C value of 26.5 was assumed to be a 100% metabolic turnover of fixed ^13^C rather than current absorption. A baseline connecting the time 0 and 120 min points was constructed by multiplying the mean delta ^13^C value at 120 min by the average cumulative increase in area under the delta ^13^C•time curve at each sampling point, from 0 at T = 0, to 100% at T = 120 min (Appendix A, Figure A5). By subtracting this baseline from the unadjusted delta^13^C curves, net delta ^13^C responses were obtained that simulated blood glucose response curves (Figure 3). The areas under the net ^13^C curves for each treatment up to 30 min, which is the time of the blood glucose peak, were calculated by trapezoid summation for comparison with the iAUCs from the blood glucose responses up to their 30 min peak. For the sake of comparison, both blood glucose and breath ^13^C were expressed as a percentage of the 30 min value for the WB+KFsug treatment (Figure 4), which was the control that contained none of the non-sugar kiwifruit components.

*Satiety*: The subjects were asked to rate their appetite at 0, 15, 60, 120 and 180 min using a four-dimension, 10 cm, visual analogue scale (VAS), with the following dimensions: How hungry do you feel? (not at all hungry—extremely hungry); How full do you feel? (not at all full—extremely full); How satisfied do you feel? (completely empty—cannot take another bite); How much food do you think you can eat right now? (nothing at all—very large amount) [17]. The area under the curve (cm.min) for each dimension was calculated.

### 2.6. Data Analysis

Participant numbers were based on our previous experience of the effect of kiwifruit on glycaemic response [6] and the fact that the current trial was intended to be a pilot study. The effect size and significance of the results confirmed that participant numbers were adequate for the study.

Incremental blood glucose responses were calculated by subtracting each individual’s baseline value from subsequent measurements (Figure 1). The incremental values were then used to determine the positive incremental area under the curve (IAUC) for each individual. GenStat software was used (version 18, VSNi Ltd., Hemel Hempstead, Unite Kingdom) in an unbalanced analysis of variance (ANOVA), testing differences between meals after adjusting for participant and order effects.

The independent effects of dietary fibre and organic acids (OA) on the outcome measures were also tested by ANOVA fitting three factors: OA, indicating presence of organic acids (not in diet 1 KFsug or diet 3 KFneut), fibre (not in diet 1 KFsug, diet 4 KFjuice, or diet 5 KFsug+OA), and KF (not in diet 1 KFsug or diet 5 KFsug+OA), plus the interaction between KF and OA (to see if the difference between KFneut and KF was similar to the difference between KFsug+OA and KFsug). The OA effect was tested adjusted for fibre; the fibre effect was tested adjusted for OA. The KF effect was tested adjusted for OA and fibre; the KF x OA interaction was tested adjusted for OA, fibre and KF. The full results of the analysis are given in Appendix A Table A2, Table A3, Table A4, Table A5, Table A6 and Table A8.

## 3. Results

**Figure A3:**
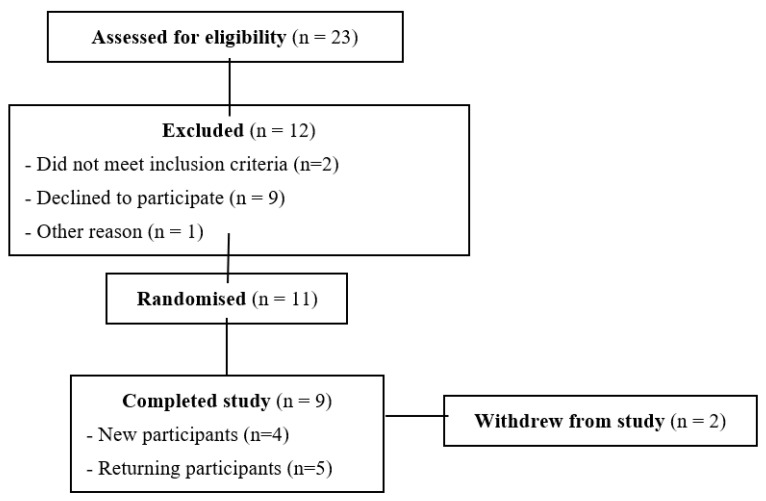
CONSORT diagram for the study of kiwifruit component effects on glycaemic response and ^13^C-acetate absorption.

**Figure A4:**
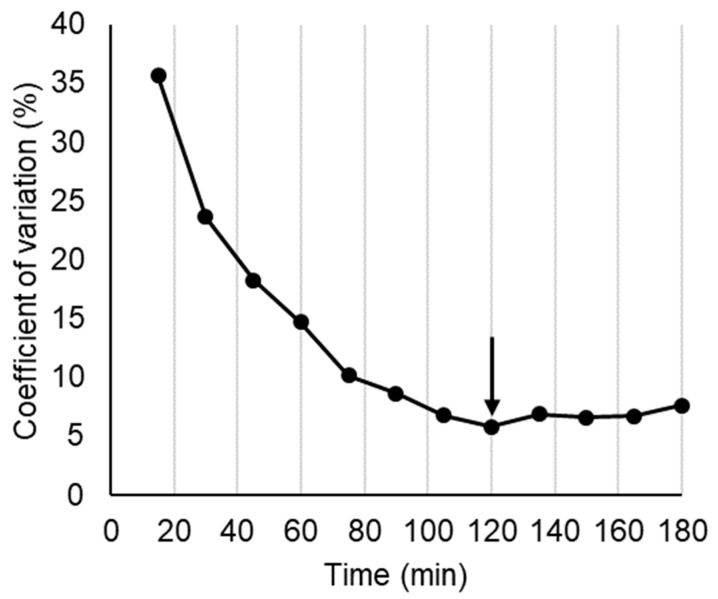
Difference between treatments in breath ^13^C as coefficient of variation between treatment means, used to estimate where differences due to treatments ended. Arrow shows point where absorption was assumed to be complete.

**Figure A5:**
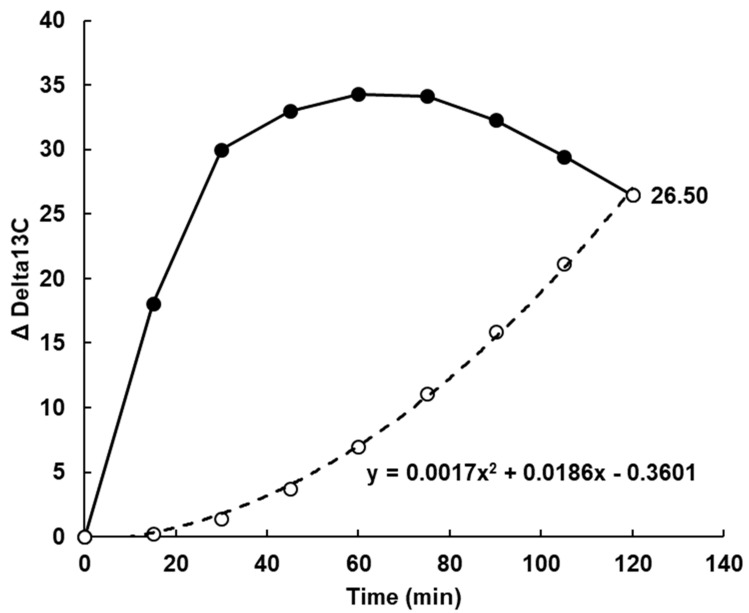
The baseline (dashed line) for ^13^C based on the cumulative area under the curve (AUC) of the mean value for all treatments at each time point (solid line). The ^13^C delta value at 100% (26.5) was multiplied by the percentage of incremental area under the curve (iAUC) accumulated, to obtain the baseline value at each time. The baseline value was subtracted from the treatment values at each time to provide a value for current absorption (Figure 3).

Eleven participants were randomised and nine completed the study (Appendix A, Figure A3, Table A1). Despite the small number of participants in this study, the results showed some very clear trends. The WB+KFsug and the WB+KF treatments were at the opposite extremes of blood glucose response amplitude and differed substantially in amplitude by 41% (*p* < 0.001) and in iAUC to 30 min by 50% (*p* < 0.001) (Table 2). All other treatments were almost intermediate between the extremes (Figure 1A and Figure 4) and up to 30 min all treatments significantly (*p* < 0.001) lowered iAUC compared with the control (Table 2). Although peak heights differed between treatments, the blood glucose iAUCs over 180 min were similar to the WB+KFsug control, except for the WB+KF treatment, which depressed iAUC by 22% compared with the control (Table 2).

The plasma insulin responses were measured in duplicate, with an average CV of 7.16%. They very closely tracked the blood glucose responses (Figure 1B, Table 3) and the iAUCs for blood glucose (x) and insulin (y) up to 30 min, as a percentage of the WB+KFsug control, correlated closely (R^2^ = 0.97, y = 1.03x − 2.3).

The ability of kiwifruit to depress glycaemic response amplitude was diminished by the removal of dietary fibre (WB+KFjuice) and by neutralising the pH from its initial value of pH 3.4 to pH 7.0 (WB+KFneut). Adding the same organic acid equivalents at the same pH as in the kiwifruit (WB+KFsug+OA) to the control (WB+KFsug) reduced glycaemic response amplitude by almost half as much as the whole kiwifruit. The effects of the dietary fibre and organic acids on response amplitude were, therefore, approximately additive.

The ^13^CO_2_ exhalation pattern was similar to that of the blood glucose responses, although the incremental ^13^CO_2_ exhalation responses based on the starting baseline extended over a much longer period than the blood glucose responses and were still well above baseline at the end of sampling at 180 min (Figure 2). While all blood glucose responses peaked at 30 min, the time of the peaks of breath ^13^CO_2_ depended on treatment (Table 4). Compared with the WB+KFsug control, the WB+KF treatment prolonged the time to peak ^13^CO_2_ exhalation by 79%, with the other treatments causing intermediate delays (Table 4).

However, with the baseline adjusted to remove background accumulation of ^13^CO_2_ over the course of the trial, the incremental (above baseline) changes in ^13^CO_2_ showed remarkably similar patterns to the blood glucose responses (compare Figure 1A and Figure 3), and differences in time to peak ^13^CO_2_ became statistically non-significant (Table 4). The post-peak descent in ^13^CO_2_ was more gradual than the post-peak blood glucose descent, so that the differences between treatments in iAUC reflected peak heights for the ^13^CO_2_ but not for the blood glucose. Averaged across treatments, 79% of the ^13^C iAUC occurred after peak, compared with 68% of the blood glucose iAUC.

Without subtraction of the cumulative background, that is, using a linear baseline from T = 0, differences in ^13^C exhalation between treatments were reduced, while the iAUC values were apparently increased and extended (Table A7). Nonetheless, the independent effects of dietary fibre and organic acids were still significant (*p* < 0.001 (Appendix A, Table A8)).

Up to 30 min post ingestion, which was the time of peak blood glucose, the cumulative iAUC for blood glucose (Table 2) and ^13^CO_2_ (Table 5) correlated closely (y = 0.75x + 25.87, R² = 0.97), showing substantial and similar effects of the treatments on rate of absorption of glucose and ^13^C acetate (Figure 4). Compared with the WB+KFsug control, whole kiwifruit (WB+KF) suppression of ^13^CO_2_ at 30 min (56%, *p* < 0.001) was close to the glycaemic response suppression (50%, *p* < 0.001) at 30 min. Neutralizing (WB+KFneut), removing fibre (WB+KFjuice) or adding organic acids (WB+KFsug+OA) significantly reduced cumulative ^13^CO_2_ exhalation at 30 min, by 35-42% (*p* < 0.001) (Table 5), and reduced blood glucose iAUC at 30 min by 28–31% (*p* < 0.001) (Table 2).

**Figure 5:**
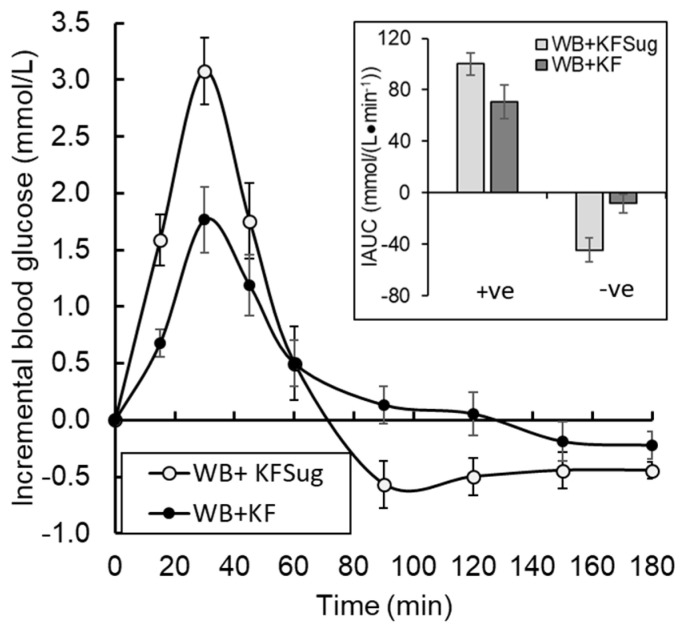
Effect of non-sugar kiwifruit components on glycaemic fluctuation as proportions of hyper- (+ve iAUC) and hypo- (-ve iAUC) glycaemia in the 180 min period following ingestion of WeetBix (WB) plus kiwifruit sugars (WB+KFsug), or WB plus whole kiwifruit (WB+KF). The curves do not include a fructose contribution to relative reduction in glycaemic response by KF, because it was excluded by the formulation (Means ± SEM).

The high blood glucose response to the WB+KFsug treatment led to a hypoglycaemic overshoot at about 75 min, which the presence of whole kiwifruit delayed until after 120 min. The overshoot was reduced by all treatments compared with the control, but particularly by the whole kiwifruit (WB+KF) (Figure 5).

Whole kiwifruit and kiwifruit components all showed signs of suppressing appetite (Table 6). Although underpowered as a study of appetite per se, the appetite suppressing effect of whole kiwifruit (WB+KF) was evident on all dimensions of appetite and significantly increased sensations of fullness (51%, *p* = 0.009) and satisfaction (44%, *p* = 0.033) over the non-kiwifruit control (WB+KFsug).

Statistical analysis of the independent effects of dietary fibre and organic acids supported the findings presented in Table 2, Table 3, Table 4 and Table 5. The full ANOVA analyses of independent effects are given in Appendix A Table A2, Table A3, Table A4, Table A5, Table A6 and Table A8. The significant independent effects of fibre and organic acids are abstracted as follows:

- Incremental blood glucose peak height (Table 2) was influenced by both OA (F, 13.4; *p* < 0.001) and fibre (F, 13.7, *p* < 0.001). AUC at 30 min was similarly influenced by OA (F, 15.4; *p* < 0.001) and fibre (F, 13.6; *p* < 0.001) (Appendix A, Table A2).
- Insulin peak height (Table 3) was influenced by OA (F, 10.9; *p* < 0.002) and fibre (F, 25.4; *p* < 0.001), and 30 min insulin AUC was also influenced by OA (F, 13.9; *p* < 0.001) and fibre (F, 19.4; *p* < 0.001). Insulin AUC to 180 min was influenced by fibre (F, 10.3; *p* < 0.003) but not OA (F, 2.1, *p* < 0.161) (Appendix A, Table A3).
- Time to the breath ^13^C peak (Table 4) without baseline adjustment was influenced by OA (F, 27.5, *p* < 0.001) and fibre (F, 15.5; *p* < 0.001). Using the adjusted baseline, time to the ^13^C peak was influenced by OA (F, 6.2; *p* < 0.002) (Appendix A, Table A4).
- The ^13^C AUC to 30 min (Table 5) was influenced by OA (F, 48.3; *p* < 0.001) and fibre (F, 18.0; *p* < 0.001), and there was a significant OA x KF interaction. The ^13^C iAUC to 120 min was influenced by OA (F, 47; *p* < 0.001) and fibre (F, 7.0; *p* < 0.012) (Appendix A, Table A5).
- Fibre influenced three of the satiety dimensions (Table 6); fullness (F, 14.6; *p* < 0.001), satisfaction (F, 9.6; *p* < 0.004) and quantity (F, 6.5; *p* < 0.016) (Appendix A, Table A6).

These results suggest that the effects of a mixed breakfast (KF+WeetBix) compared to WeetBix alone are mostly due to the OA and Fibre components in KF. Fibre is a bigger influence on insulin and satiety than OAs, which are a bigger influence on ^13^C results. A mixed model analysis gave the same results.

## 4. Discussion

The present study has confirmed earlier observation that non-sugar kiwifruit components have the capacity to significantly suppress glycaemic response amplitude [6], most evident in the difference of 50% (*p* < 0.05) between the kiwifruit-free control (WB+KFsug) and the treatment containing whole kiwifruit (WB+KF). We have extended this observation by showing that the effect is due to dietary fibre and organic acids acting equally. By including sodium [1-^13^C] acetate as a reference, we have shown that the observed effects probably result from kiwifruit components reducing the rate of absorption from the gut. Removing about 75% of the dietary fibre in the WB+KFjuice treatment halved the suppression of glycaemic response amplitude by KF. Adjusting the kiwifruit pH from 3.4 to 7.0 had a similar-sized effect. Conversely, adding organic acids to the WB+KFsug achieved about half of the suppression of glycaemic response caused by whole kiwifruit. Therefore, it appears that both cell walls and organic acids have an important role to play in kiwifruit reducing the amplitude of the glycaemic response to co-consumed cereals. Furthermore, the reductions measured in the research reported here are in addition to those that would be obtained by substituting fruit sugar for starch, in an equicarbohydrate exchange. The present study was designed to exclude differences between treatments in carbohydrate amount and type, avoiding the fructose effect and allowing non-confounded measurement of the effect of non-sugar components. The effects of organic acids and dietary fibre were very clear cut, despite the limitation of being based on finger prick rather than venous blood analysis.

Adding dietary fibre to fruit juice has been shown to decrease glycaemic response [18]. A role for organic acids in suppressing glycaemic response is also well established [9]. The present research has shown that both of these effects have approximately equal and complementary roles in the antiglycaemic action of kiwifruit consumed in a carbohydrate exchange format. The parallel effects on breath ^13^CO_2_ from co-ingested acetate also strongly indicate that the antiglycaemic effect of the non-sugar components of kiwifruit is not due to phytochemical micro-components inhibiting glucose uptake, because most of the effect could be accounted for by the sum of the macro-component effects of dietary fibre and organic acids on absorption from the gut. Moreover, the similarity of the effects on blood glucose and breath ^13^CO_2_ suggests that specific glucose uptake inhibitors did not play a role in retarding glucose uptake.

Because the effect of dietary fibre in this study was determined by comparing a full-fibre (control) with a fibre-reduced (kiwifruit juice) treatment, rather than with a completely fibre-free treatment, the effect of fibre may have been slightly underestimated. However, using minimally processed, cold-pressed juice as a reduced-fibre treatment avoided the problem of degradation and extraction of pectin, which is a substantial component of fruit cell walls. An alternative approach, that of including purified isolated kiwifruit fibre within a meal formulation, runs the risk of a change in fibre properties altering gut level effects as compared with intrinsic fibre, so that effects could be invalid or missed altogether. Although not perfect, the fibre-reduced cold pressed juice option used in the present study gave a valid indication of the role of dietary fibre, which can be explored in further research.

The observation that neutralising the kiwifruit reduced the suppression of glycaemia suggests that the organic acid effect was a pH effect rather than a metabolic effect of absorbed organic acids. The organic acid treatment (WB+SAug+OA) was at the pH of kiwifruit (pH 3.3). The same treatment after neutralising would be interesting to include to see whether neutralised organic acids had any effect on their own.

The sodium [1-^13^C] acetate reference, ingested in all treatments in this study, was used to determine gastric emptying through measurement of breath ^13^CO_2_. However, as sodium acetate is absorbed from the intestine, not the stomach, the appearance of the ^13^CO_2_ will be subject to many of the same gut-level factors, involving the stomach and intestine, which delay glucose uptake, lowering glycaemic response. Such factors could include a combination of reduced gastric emptying rate, reduced gastric and duodenal mixing, reduced glucose diffusion, reduced gastric starch digestion due to low pH of the kiwifruit and organic acids [19], and reduced duodenal digestion due to the physical properties of the duodenal chyme.

Using breath ^13^CO_2_ to determine the effects of diets on intestinal absorption in a way that allows direct comparison with the effects on blood glucose is not completely straight forward [20]. Blood glucose is a relatively direct measure of glucose uptake, while breath CO_2_ is a less direct measure of acetate uptake, as it relies on prior metabolic oxidation. Absorbed acetate is rapidly converted by acyl-CoA short-chain synthases to acetyl coenzyme A, which is a basic feedstock for carbon metabolism [21]. While about 50–70% of the acetate is estimated to be quickly converted to CO_2_ by the Krebs cycle and exhaled [22], the rest is redistributed and retained throughout the body in the bicarbonate pool and other carbon sinks, where it is subjected to more delayed metabolic turnover, appearing as a cumulative rise in baseline breath ^13^CO_2_. However, assuming that the ratio of immediately respired to “fixed” acetate remains constant, the breath ^13^CO_2_ should be directly proportional to the amount of ^13^C-acetate absorbed.

In the present study, we attempted to isolate the ^13^C response that most immediately reflected treatment effects on absorption, by removing the delayed effects of metabolic turnover of ^13^C that had already been assimilated beyond the Krebs cycle. A baseline was constructed to separate “current” from “historical” absorption by subtracting the delayed ^13^C metabolism, estimated by multiplying the ^13^C delta value at 120 min by the percentage of the cumulative delta-^13^C iAUC aggregated at each time point up to an assumed value of 100% at 120 min. At 120 min, any differences due to absorption were obscured by background. With the baseline subtracted, the net delta ^13^C responses were very similar to the glycaemic responses, although they were broader and parabolic (Figure 3), whereas the post-peak declines in blood glucose were more rapid and linear. Furthermore, differences in time to peak were substantially reduced by removing the delayed ^13^C release.

The difference between blood glucose and net delta-^13^C in the shape of the post-peak curves can be attributed to the acute postprandial metabolic correction needed to maintain blood glucose concentrations within narrow physiological limits, which would tend to compress and reduce differences between treatments in blood glucose responses to them. Acetate, as a minor component of the diet, is not subjected to such urgent postprandial regulation. Postprandial glycaemia thus becomes a transient phenomenon under strict homeostatic control, curtailed by the insulin and associated responses well before gastric emptying and carbohydrate absorption is complete [23]. The parameters used in studies of gastric emptying were, therefore, not appropriate or necessary for this study.

Because intense blood glucose regulation compresses the blood glucose response curve, the net delta-^13^C curves (Figure 3) probably give a more accurate indication of the effect of gut-level factors on glucose absorption than the complete blood glucose response curves themselves. Therefore, in the present investigation ^13^C-acetate was used to explore absorption specifically in the 30 min timeframe of blood glucose loading up to the peak postprandial response, before the insulin response had gained full momentum.

A single baseline to adjust ^13^C values for all treatments was an approximation. In fact, each treatment will have its own baseline, dependent on the rate of acetate loading; however, an overall baseline, as calculated here, was sufficient adjustment within the 30 min period in which intestinal absorption leading to peak glycaemic response occurred, and within which the contribution of the cumulative baseline was still relatively small. Furthermore, the close correspondence (R^2^ = 0.97; Figure 4) between the effects of the treatments on ^13^C and blood glucose provided an internal concurrent validation of the analysis. Shifting the baseline to 20.86 at 150 min increased the % WB+KFsug values in Figure 4 by only 3%.

The effects of the kiwifruit treatments on peak height were greater than the effects on AUC. There is growing evidence that lowering glycaemic response amplitude is an important factor in limiting the widespread systemic damage and elevated risk of cardiovascular events [24] and cerebral decline [25] caused by hyperglycaemia. Secondary processes involved in diabetic pathology, such as the formation of advanced glycation end products, oxidative stress, chronic inflammation and endothelial dysfunction have been linked to glycaemic excursions [26] involving both hyper- and hypo-glycaemic fluctuations [25]. Therefore, even without a large effect on the overall blood glucose response AUC, substantially lowering postprandial response amplitude and reducing subsequent hypoglycaemia may together confer dietary protection against the numerous health complications associated with diabetes. The clear ability of kiwifruit to suppress glycaemic fluctuation (Figure 5) suggests that they have a place in diets aimed at averting the effects of long-term exposure to glycaemia, as part of maintaining good health. However, the present study suggests that fruit would be most beneficial in glycaemia management if it were consumed as part of, or as a preload to, a meal, so that the organic acid and cell wall components of the fruit are able to interact with the glycaemic carbohydrates from other diet components. In associated work, we found that glycaemic response amplitude was suppressed by about 20% if kiwifruit were consumed 30 min after a wheat biscuit, but by about 48% if kiwifruit were consumed 30 min before the wheat biscuit (to be published).

Apart from the direct benefits of moderating and stabilising blood glucose concentrations, additional health benefits may arise from the suppression of insulin demand and insulin response, which are well known to be a function of blood glucose concentrations and which have their own cluster of associated risk factors for disease [27,28].

The ability of whole kiwifruit to reduce the postprandial glycaemic peak, avert postprandial hypoglycaemia, and maintain blood glucose at slightly above baseline for an extended period (Figure 5) could have numerous benefits that stem from improved glucose regulation in healthy individuals. Subjective energy, or “vitality”, is sensitive to blood glucose [29]. Averting hypoglycaemia in the late post-prandial period has been shown to maintain cognitive capacity [30].

Although the present pilot study was underpowered as a study of appetite, the consistent difference between the kiwifruit-free (WB+KFsug) and whole kiwifruit (WB+KF) treatments (Table 6) in dimensions of satiety suggested an appetite-suppressing effect of the kiwifruit, previously noted as a delay in onset of hunger [6]. The satiety findings are consistent with the putative roles of blood glucose and insulin in appetite control [31] and are relevant to obesity. They suggest that as well as reducing postprandial glycaemia and insulinaemia, kiwifruit may be used in glycaemia management to reduce the tendency to over-snack between meals or overeat at subsequent meals. The trends observed in the present study indicate that a statistically and quantitatively significant effect of kiwifruit on appetite would be revealed in a larger study designed specifically to test appetite as a primary outcome.

The number of subjects who completed the present study was small (N = 9), but not unusually so, for many studies of effects of foods and food components on glycaemic response use less than 10 subjects [32], which is the number recommended for glycaemic index determination [33]. In the present study the effect sizes and their statistical significance suggest the study was sufficiently powered to separate the effect of individual components from the effects of both the whole fruit and the fruit-free control. However, it would be valuable to extend the research in further studies with larger subject numbers.

When the anti-glycaemic effects of fructose substitution of highly digestible starch are added to the effects of non-sugar components, demonstrated in the present study, appreciable glycaemic benefits can be expected from using whole kiwifruit flesh to partially substitute starchy foods in carbohydrate exchange. As kiwifruit are unlikely to be unique amongst fruit, the results can probably be generalised to the use of other fruit in prudent diets for glycaemia management—a subject demanding further research.

## 5. Conclusions

The ability of kiwifruit to suppress the glycaemic response to co-consumed starch is governed not only by fructose substitution of highly glycaemic starch, but also by the dietary fibre and organic acid components of the kiwifruit retarding glucose uptake from the small intestine. The research presented here suggests that ingestion of the whole edible portion of kiwifruit would provide greater glycaemic benefit than fractionated kiwifruit products, because all of the major kiwifruit components—low glycaemic index sugars, dietary fibre and organic acids—contribute to reducing blood glucose response during carbohydrate exchange.

## Figures and Tables

**Table 1:** Composition of meals to test the effect of kiwifruit organic acids, pH and dietary fibre on gastric emptying and on glycaemic response to available carbohydrate.

Meal	1	2	3	4	5
Treatment	WB+ KFsug	WB+ KF	WB+ KFneut	WB+ KFjuice	WB+KFsug +OA
Diet composition	WeetBix™	Kiwifruit pH 3.3	Kiwifruit pH 7	Kiwifruit juice pH 3.3	Organic acids pH 3.3
WeetBix™ (g)	30	30	30	30	30
Kiwifruit flesh (g)	-	200	200	-	-
Kiwifruit juice (mL)	-	-	-	194	-
2.M NaOH (mL)	-	-	20.7	-	-
Kiwifruit sugars ^1^ (g)	20.4	-	-	-	20.4
Organic acids ^2^ (mL)	-	-	-	-	50
^13^C acetate (mL)	1.5	1.5	1.5	1.5	1.5
Water (mL)	200	20.4	-	26	150
Total	251.9	251.9	252.2	251.5	251.5

WeetBix™: Carbohydrate 67 g/100 g. For 20 g carbohydrate, require 29.85 g WeetBix™. ^1^ Glucose:fructose:sucrose (2:2:1). ^2^ Citric acid, 35.3 g, malic acid 9.46 g, ascorbic acid 2.55 g, adjusted to pH 3.3 in 750 mL water.

**Table 2:** Influence of kiwifruit components on incremental blood glucose peak height (iBGRmax), and incremental area under the blood glucose response curve at 30 min (iAUC_30_) and 180 min (iAUC_180_) compared with the kiwifruit-free sample (WB+KFsug).

	iBGRmax (mmol/L)			∑iAUC_30_ (mmol/L∙min)			∑iAUC_180_ (mmol/L∙min)		
	Mean	SEM	Change in Mean (%)	Mean	SEM	Change in Mean (%)	Mean	SEM	Change in Mean (%)
WB+KFsug	3.08 ^c^	0.29	0	46.9 ^c^	5.3	0.0	109.6	11.6	0.0
WB+KF	1.80 ^a^	0.26	−41.6	23.4 ^a^	3.8	−50.1	85.0	16.7	−22.3
WB+KFneut	2.49 ^b^	0.31	−19.2	33.5 ^b^	4.9	−28.6	117.0	14.9	6.4
WB+KFjuice	2.39 ^b^	0.29	−22.3	32.6 ^ab^	4.0	−30.5	109.7	17.6	0.4
WB+KFsug+OA	2.59 ^bc^	0.18	−15.9	34.0 ^b^	3.8	−27.5	115.3	14.4	5.5
SE	0.19			3.3			11.1		
ANOVA									
Diet F (4 and 32 df)	6.1			6.3			1.4		
P	<0.001			<0.001			0.273		

Means in a column that do not have the same superscript letter differ significantly.

**Table 3:** Influence of non-sugar kiwifruit components on insulin incremental peak height and on insulin iAUC at 30 min (iAUC_30_) and 180 min (iAUC_180_) compared with the kiwifruit-free control sample (WB+KFsug).

	Peak Height (μU/mL)			iAUC_30_ (μU/mL∙min)			iAUC_180_ (μU/mL∙min)		
Diet	Mean	SEM	Change in Mean (%)	Mean	SEM	Change in Mean (%)	Mean	SEM	Change in Mean (%)
WB+KFsug	103 ^c^	12.3	0.0	1804 ^b^	224	0	4745 ^b^	670	0.0
WB+KF	57 ^a^	5.6	−44.7	872 ^a^	83	−52	3499 ^a^	433	−26.3
WB+KFneut	68 ^a^	9.2	−34.0	1228 ^ab^	164	−32	4080 ^ab^	661	−14.0
WB+KFjuice	73 ^ab^	8.6	−29.1	1247 ^ab^	79	−31	4142 ^ab^	550	−12.7
WB+KFsug+OA	86 ^bc^	7.5	−16.5	1401 ^b^	140	−22	4872 ^b^	375	2.7
SE	6			290			290		
ANOVA									
Diet F (4 and 32 df)	8.9			7.4			3.7		
*p*	<0.001			<0.001			0.014		

Means in a column that do not have the same superscript letter differ significantly.

**Table 4:** Time to peak ^13^CO_2_ exhalation compared with the control (WB+KFsug) before and after adjusting for cumulative increase in ^13^C baseline.

	Time to Peak before Adjusting			Time to Peak after Adjusting		
	Mean (min)	SEM	Change in Mean (%)	Mean (min)	SEM	Change in Mean (%)
WB+KFsug	48.3 ^a^	6.0	0	36.7	2.6	0
WB+KF	86.7 ^c^	4.2	79	45.0	4.3	23
WB+KFneut	70.0 ^b^	6.1	45	43.3	3.9	18
WB+KFjuice	68.3 ^b^	4.4	41	50.0	3.5	36
WB+KFsug+OA	80.0 ^bc^	3.5	66	50.0	4.3	36
SE	4.5			3.6		
ANOVA						
Diet F (4 and 32 df)	10.4			2.3		
*p*	<0.001			0.076		

Means in a column that do not have the same superscript letter differ significantly.

**Table 5:** Incremental area under the Delta ^13^C-time curve to 30 and 120 min compared with the control (WB+KFsug), after adjusting for cumulative increase in ^13^C baseline.

	∑iAUC to 30 min			∑iAUC to 120 min		
	Mean (^13^C)	SEM	Change in Mean (%)	Mean (^13^C)	SEM	Change in Mean (%)
WB+KFsug	722 ^c^	71	0	2838 ^b^	246	0
WB+KF	318 ^a^	38	−56	1676 ^a^	246	−41
WB+KFneut	473 ^b^	57	−35	2471 ^b^	253	−13
WB+KFjuice	417 ^b^	39	−42	2032 ^a^	172	−28
WB+KFsug+OA	462 ^ab^	51	−36	2398 ^a^	243	−15
SE	39			132		
ANOVA						
Diet F (4 and 32 df)	16.0			12.5		
*p*	<0.001			<0.001		

Means in a column that do not have the same superscript letter differ significantly.

**Table 6:** Effect of whole kiwifruit and kiwifruit components on dimensions of satiety measured on a visual analogue scale, expressed as area under the curve (cm∙min) of measurements at 0, 15, 60, 120 and 180 min after ingestion. Means in a column that do not have the same superscript letter differ significantly.

	Hunger			Fullness			Satisfaction			Quantity		
Diet	Mean AUC	SEM	Change in Mean (%)	Mean AUC	SEM	Change in Mean (%)	Mean AUC	SEM	Change in Mean (%)	Mean AUC	SEM	Change in Mean (%)
WB+KFsug	690	155	0	708 ^a^	105	0	717 ^a^	107	0	846	124	0
WB+KF	572	102	−17	1070 ^c^	106	51	1029 ^b^	120	44	710	156	−16
WB+KFneut	648	143	−6	949 ^bc^	120	34	880 ^ab^	113	23	767	156	−9
WB+KFjuice	686	115	−1	801 ^ab^	117	13	776 ^a^	84	8	859	148	2
WB+KFsug+OA	576	122	−17	840 ^ab^	102	19	840 ^ab^	100	17	809	148	−4
SE	73			69			68			44		
ANOVA												
Diet F (4 and 32 df)	0.6			4.1			3			1.9		
*p*	0.647			0.009			0.033			0.136		

## Data Availability

Data may be requested from the corresponding author (J.M.).

## Appendix A

*Recruiting**and screening:* The volunteers were asked initial recruitment questions in order to determine their suitability to take part in the study. The nature of the study and their involvement and responsibilities were described to them. Volunteers deemed suitable were sent an information sheet containing study details and an informed consent form. Volunteers who were willing to participate were then called to the clinic. A blood sample was taken to measure fasting blood glucose and HbA1c. Anthropometric measurements were also taken (Appendix A Table A1). This first visit was also used to familiarise participants with the blood sampling procedure they would be subjected to as volunteers in the study. The health of the volunteers was gauged by self-assessment and by their scores on the General Health Questionnaire.

**Table A1:** Anthropometric data for the participants.

Age (y)	18–40
Gender	6 female, 3 male
Ethnicity	Mixed
Smoke	None
Diabetes	None
Blood pressure (mm Hg)	92/56–134/78
Weight, mean ± SD (range) (kg)	73.8 ± 16 (52–105)
Height, mean ± SD (range) (m)	1.68 ± 0.05 (1.59–1.74)
Body mass index (BMI), mean ± SD (range) (kg/m^2^)	25.9 ± 4.5 (20.6–32.9)
Fasting blood glucose, mean ± SD (range) (mmol/L)	4.7 ± 0.45 (4.1–5.4)
HbA1c (mmol/mol)	32.7 ± 3.5 (29–40)

**Table A2:** Factors influencing incremental blood glucose response (iBGR) and area under the iBGR curve (AUC) determined by analysis of variance of within-person effects (means are in Table 2; significant effects are bolded).

		iBGR Max		iBGR AUC to 30 min		iBGR AUC to 180 min	
Source of Variation	d.f.	F	*p*	F	*p*	F	*p*
OA *	1	**13.4**	**<0.001**	**15.4**	**<0.001**	1.4	0.250
Fibre *	1	**13.7**	**<0.001**	**13.6**	**<0.001**	1.5	0.231
KF	1	0.3	0.561	0.2	0.645	0.1	0.795
OA.KF	1	0.3	0.595	0.2	0.669	2.9	0.099
Residual	32						

* Each effect adjusted for the other.

**Table A3:** Factors influencing insulin response peak height and area under the insulin response curve determined by analysis of variance of within-person effects (means are in Table 3; significant effects are bolded).

		Insulin Peak Height		Insulin AUC to 30 min		Insulin AUC to 180 min	
Source of Variation	d.f.	F	*p*	F	*p*	F	*p*
OA *	1	**10.9**	**0.002**	**13.9**	**<0.001**	2.1	0.161
Fibre *	1	**25.4**	**<0.001**	**19.4**	**<0.001**	**10.3**	**0.003**
KF	1	3.6	0.066	1.0	0.32	2.1	0.16
OA.KF	1	0.3	0.599	0.0	0.85	1.5	0.232
Residual	32						

* Each effect adjusted for the other.

**Table A4:** Factors influencing time to peak ^13^C exhalation with and without subtraction of cumulative baseline ^13^C, determined by analysis of variance of within-person effects (means are in Table 4; significant effects are bolded).

		Time to Peak Without Baseline Subtraction		Time to Peak After Baseline Subtraction	
Source of Variation	d.f.	F	*p*	F	*p*
OA *	1	**27.5**	**<0.001**	**6.2**	**0.018**
Fibre *	1	**15.5**	**<0.001**	0.0	1.000
KF	1	1.8	0.194	0.4	0.546
OA.KF	1	2.8	0.106	2.6	0.116
Residual	32				

* Each effect adjusted for the other.

**Table A5:** Factors influencing time to peak ^13^C exhalation with and without subtraction of cumulative baseline ^13^C, determined by analysis of variance of within-person effects (means are in Table 5; significant effects are bolded).

		^13^C iAUC to 30 min		^13^C iAUC to 120 min	
Source of Variation	d.f.	F	*p*	F	*p*
OA *	1	**48.3**	**<0.001**	**47.0**	**<0.001**
Fibre *	1	**18.0**	**<0.001**	**7.0**	**0.012**
KF	1	0.0	0.852	0.3	0.597
OA.KF	1	**5.7**	**0.023**	0.3	0.601
Residual	32				

* Each effect adjusted for the other.

**Table A6:** Factors influencing dimensions of satiety determined by analysis of variance of within-person effects (means are in Table 6; significant effects are bolded).

Source of Variation		Hunger		Fullness		Satisfaction		Quantity	
	d.f.	F	*p*	F	*p*	F	*p*	F	*p*
OA *	1	1.0	0.331	3.3	0.08	3.4	0.076	0.6	0.449
Fibre *	1	0.6	0.448	**14.6**	**<0.001**	**9.6**	**0.004**	**6.5**	**0.016**
KF	1	1.1	0.305	0.2	0.697	0.6	0.439	0.9	0.357
OA.KF	1	0.1	0.791	0.0	0.942	0.0	0.851	0.1	0.82
Residual	32								

* Each effect adjusted for the other.

**Table A7:** Area under the Delta ^13^C ● Time curve (iAUC) without adjusting for cumulative increase in baseline.

	iAUC to 30 min			iAUC to 120 min			iAUC to 180 min		
	Mean iAUC	SEM	Change in Mean (%)	Mean iAUC	SEM	Change in Mean (%)	Mean iAUC	SEM	Change in Mean (%)
WB+KFsug	1248 ^c^	118	0	3918 ^c^	247	0	5085 ^b^	333	0
WB+KF	570 ^a^	66	−54	2756 ^a^	246	−30	4042 ^a^	354	−21
WB+KFneut	836 ^b^	94	−33	3551 ^bc^	253	−9	4901 ^b^	341	−4
WB+KFjuice	736 ^ab^	66	−41	3112 ^a^	172	−21	4293 ^a^	232	−16
WB+KFsug+OA	816 ^b^	85	−35	3505 ^b^	235	−11	4825 ^b^	294	−5
SE	64			132			171		
ANOVA									
Diet F (4 and 32 df)	15.2			11.4			6.7		
*p*	<0.001			<0.001			<0.001		

Means in a column that do not have the same superscript letter differ significantly.

**Table A8:** Factors influencing incremental area under the ^13C^ exhalation curve to 30 min and 120 min without subtraction of cumulative baseline (means are in Table A7; significant effects are bolded).

Source of Variation		^13^C iAUC to 30 min		^13^C iAUC to 120 min		^13^C iAUC to 180 min	
	d.f.	F	*p*	F	*p*	F	*p*
OA *	1	**41.5**	**<0.001**	**31.9**	**<0.001**	**17.9**	**<0.001**
Fibre *	1	**24.5**	**<0.001**	**15.0**	**<0.001**	**5.6**	**0.024**
KF	1	2.0	0.164	2.9	0.098	2.9	0.100
OA.KF	1	1.7	0.208	2.1	0.158	3.1	0.089
Residual	32						

* Each effect adjusted for the other.
